# Cancer-Related Cachexia: The Vicious Circle between Inflammatory Cytokines, Skeletal Muscle, Lipid Metabolism and the Possible Role of Physical Training

**DOI:** 10.3390/ijms23063004

**Published:** 2022-03-10

**Authors:** Giuseppe Donato Mangano, Malak Fouani, Daniela D’Amico, Valentina Di Felice, Rosario Barone

**Affiliations:** Department of Biomedicine, Neuroscience and Advanced Diagnostics (BIND), University of Palermo, 90127 Palermo, Italy; malak.fouani@unipa.it (M.F.); damicoda90@gmail.com (D.D.); valentina.difelice@unipa.it (V.D.F.)

**Keywords:** cancer-related cachexia, skeletal muscle, physical training, inflammatory cytokines

## Abstract

Cachexia is a multifactorial and multi-organ syndrome that is a major cause of morbidity and mortality in late-stage chronic diseases. The main clinical features of cancer-related cachexia are chronic inflammation, wasting of skeletal muscle and adipose tissue, insulin resistance, anorexia, and impaired myogenesis. A multimodal treatment has been suggested to approach the multifactorial genesis of cachexia. In this context, physical exercise has been found to have a general effect on maintaining homeostasis in a healthy life, involving multiple organs and their metabolism. The purpose of this review is to present the evidence for the relationship between inflammatory cytokines, skeletal muscle, and fat metabolism and the potential role of exercise training in breaking the vicious circle of this impaired tissue cross-talk. Due to the wide-ranging effects of exercise training, from the body to the behavior and cognition of the individual, it seems to be able to improve the quality of life in this syndrome. Therefore, studying the molecular effects of physical exercise could provide important information about the interactions between organs and the systemic mediators involved in the overall homeostasis of the body.

## 1. Introduction

Cachexia is a multifactorial and multi-organ syndrome that is a major cause of morbidity and mortality in late-stage chronic diseases such as acquired immune deficiency syndrome (AIDS), chronic obstructive pulmonary disease (COPD), congestive heart failure (CHF), multiple sclerosis, tuberculosis, and cancer [1]. The prevalence of cachexia is high, ranging from 5% to 15% in CHF or COPD to 60% to 80% in advanced cancer [2]. Half of all cancer deaths worldwide (~8.2 million people per year) are due to the cancers most commonly associated with cachexia, such as pancreatic (0.33 million deaths), esophageal (0.40 million), gastric (0.72 million), pulmonary (1.59 million), hepatic (0.75 million), and colorectal (0.69 million) cancers [3]. According to Fearon [4], cancer cachexia is a spectrum consisting of three clinically relevant stages: pre-cachexia, cachexia, and refractory cachexia. The proposed diagnostic criteria are: weight loss >5% in the past 6 months (excluding simple starvation); or body mass index (BMI) <20 and any degree of weight loss >2%; or an appendicular skeletal muscle index consistent with sarcopenia (men <7·26 kg/m^2^; women <5·45 kg/m^2^) and any degree of weight loss >2%. The main clinical features of cancer-related cachexia are chronic inflammation, wasting of skeletal muscle and adipose tissue, insulin resistance, anorexia, and impaired myogenesis [5].

Consistent with the hypermetabolic state leading to adipose tissue and skeletal muscle wasting, considerable interest has focused on the role of pro-inflammatory cytokines in mediating cachexia [6]. Cytokines imbalance directly affects the molecular mechanism underlying muscle growth but also acts indirectly by disrupting hypothalamic mechanisms that control energy-wasting, hunger, and satiety. Thus, a multimodal treatment for cancer-related cachexia has been suggested [7]. In this context, exercise training is currently recommended for the clinical management of low inflammatory diseases such as cancer cachexia, as its overall efficacy seems to offset the impairment of skeletal muscle function and attenuate muscle mass loss and fat metabolism. The purpose of this review is to present the evidence for the relationship between inflammatory cytokines, skeletal muscle, and fat metabolism and the potential role of exercise training in breaking the vicious circle of this impaired tissue cross-talk.

## 2. Cellular Processes Involved in Muscle Mass Homeostasis

The maintenance and modulation of skeletal muscle mass are attributed to two processes: protein turnover and myonuclear turnover, which are in homeostatic balance. In skeletal muscle wasting, the balance between these two processes shifts toward inhibition of muscle protein synthesis (MPS), activation of muscle protein breakdown, and reduction in myonuclear growth.

**Figure 1:**
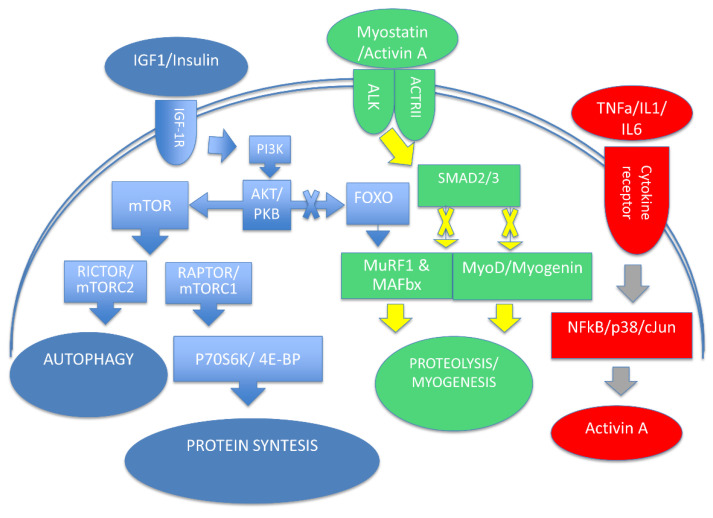
Maintenance and modulation of skeletal muscle mass are attributed to protein turnover. In blue, the insulin growth factor-1(IGF-1)–phosphoinositide−3−kinase (PI3K)–Akt/protein kinase B (PKB)–mammalian target of rapamycin (mTOR) pathway leading to protein synthesis. In red, the signaling pathway activated by myostatin that inhibits protein synthesis and reduces myotube size, leading to muscle wasting. In green, the role of inflammatory cytokines in protein degradation.

Protein turnover is a dynamic process determined by protein synthesis and degradation and mediated by transcriptional, translational, and posttranslational mechanisms [8]. An important regulatory circuit of protein turnover is the insulin growth factor-1(IGF-1), phosphatidylinositol-4,5-bisphosphate 3-kinase (PI3K)/protein kinase B (Akt), and the mammalian target of rapamycin (mTOR) pathway [9,10]. Among the different isoforms of Akt, Akt2 form is mainly expressed in skeletal muscle representing a key mediator that stimulates protein synthesis through the activation of mTOR, which forms two distinct complexes, known as the target of rapamycin complex 1/2 (TORC1 and TORC2) [11]. The TORC1 requires an adaptor protein known as the regulatory-associated partner of TOR (RAPTOR), which is necessary for normal muscle maintenance. TORC1 signals to the p70S6 kinase (S6K) and 4E-binding protein (4E-BP) pathway, which stimulates ribosome formation and protein synthesis, while TORC2 signaling appears to control autophagy, which also plays a role in muscle maintenance [12]. It has been reported that S6K appears to be involved in translational efficiency/capacity through the addition of myonuclei [13]. In addition to mTOR, Akt-mediated phosphorylation of glycogen synthase kinase-3β (GSK3β) is another critical downstream signaling pathway of IGF-1. As reported in hypertrophic muscle conditions, GSK3β is phosphorylated and its activity is inhibited, leading to activation of the eukaryotic translation initiation factor 2B (eIF2B) and the transcriptional activator β-catenin [14,15]. On the other hand, muscle mass maintenance is also characterized by proteolytic processes. The positive effect of IGF-1 on muscle growth is counteracted by myostatin. As shown in differentiated myotubes and animal models, myostatin inhibits protein synthesis and decreases myotube size, leading to muscle wasting [16,17]. Myostatin is synthesized and secreted primarily by skeletal muscle cells and signals through the activin-type II receptor (ActRII) [18]. ActRII recruits an Alk family kinase, leading to the activation of the small mother against decapentaplegic (Smad) 2/3 transcription factor complex that mediates inhibition of genes associated with muscle differentiation [19]. A study by Conery and colleagues documented that activation of Akt downregulates ActRIIB, blocking the atrophy-inducing effects of the constitutively active activin receptor-like kinase (ALK) 4/5 [20]. This study suggests a complex interplay between Akt-mTOR signaling and myostatin/Smad signaling pathways (Figure 1).

Other processes that highly regulate protein turnover are proteolytic enzyme systems, including the ubiquitin-proteasome system (UPS), the autophagy-lysosomal pathway (ALP), caspases, and calpains. As reported by Khalil [21], the UPS consists of an enzymatic cascade, involving ubiquitin-activating enzymes (E1), conjugating enzymes (E2), and ligating enzymes (E3), which culminates in the activity of 26S proteasome complexes. Experimental studies in muscle RING finger 1 (MuRF1) and muscle atrophy F-box (MAFbx)/atrogin-1 knock out mice and C2C12 myotubes have shown that deletion of MuRF1 leads to degradation of myofibrillar proteins, suggesting the prominent role of these E3-Ub ligases in muscle wasting [22,23]. An interesting study by Sandri and colleagues reported a key role of the forkhead box transcription factors (FOXO) inducing MAFbx/atrogin-1 [24]. They reported that constitutively active FOXO3 acts on the atrogin-1 promoter to cause atrogin-1 transcription leading to atrophy of myotubes and muscle fibers. Interestingly, studies of C2C12 muscle cell lines treated with dexamethasone (DEX) revealed a dose-dependent increase in MAFbx and MuRF1 transcripts leading to myotube protein degradation and reduction in myotube diameter. Moreover, the constitutive expression of the PI3K/Akt active forms in C2C12 lines was able to stop the upregulation of both DEX-induced MAFbx and MuRF1 transcripts. In particular, the authors demonstrated that Akt inactivates FOXO via a phosphorylation process, preventing its nuclear translocation. Thus, Akt is not only involved in prosynthetic pathways, but it can also prevent muscle atrophy, suppressing catabolic pathways [25].

It is known that, in cachexia, chronic inflammation leads to increased catabolism in peripheral tissues associated with mitochondrial dysfunction in skeletal muscle [26]. In cancer patients and animal models of cancer cachexia, alterations in mitochondrial structure and function have been reported, including large sarcomeric mitochondria, branched intermyofibrillar mitochondria, and sarcomere disintegration [27,28,29]. Interestingly, in the mouse model of Lewis lung carcinoma, muscle mass atrophy occurs following mitochondrial alterations [27]. A study by Brown and colleagues suggests that an increase in mitochondrial oxidative stress is followed by degeneration of the mitochondrial network and that loss of mitochondrial function is the final step before the first signs of muscle wasting [30]. Functionally, damaged mitochondria result from mitochondrial fission and are removed by mitophagy [31]. In a recent review addressing the relationship between changes in the autophagy–lysosome pathway and the occurrence of myopathies, increased levels of LC3-II along with an accumulation of receptor p62 were suggested to be markers of autophagic flux [32].

Thus, the autophagic process involved in muscle homeostasis is important in removing damaged cell organelles and misfolded protein aggregates and preventing the accumulation of toxins. Moreover, autophagy is important during muscle regeneration to regulate the balance between myonuclear growth and apoptosis, thus acting on satellite cells (SCs), the local progenitor cells of skeletal muscle [8]. It is well known that myoblasts proliferate by asymmetric cell division into two populations: one of them returns to the resting state as progenitor cells, while the other differentiates and fuses with muscle fibers.

At the molecular level, Bentzinger et al. reported that several myogenic regulators modulate the step of proliferation and differentiation [33]. In particular, the myogenic determination gene (MyoD) was associated with proliferation and early differentiation steps while MyoG seems to exert its function in late differentiation, fusion, and myotube formation.

Finally, other proteolytic enzyme systems include calpains and caspases, which represent a family of calcium-dependent and cysteine proteases, respectively. It has been reported that caspase-3 can cooperate with calpain to initiate the degradation of actin, myosin, and other structural proteins [34].

## 3. Evidence for Inflammation as a Therapeutic Target in Cachexia

Systemic inflammation is currently considered the most important cause of muscle wasting in cancer-related cachexia. Functional changes in brain regions regulating energy homeostasis contribute to the occurrence of anorexia, decreased food intake, and increased loss of muscle mass and adipose tissue [35]. Consequently, inflammatory markers are commonly used as predictors not only of metabolic abnormalities, but also of clinical outcomes [36].

Several in vitro and in vivo studies have shown that proinflammatory cytokines play a role in muscle wasting related syndromes such as cancer-related cachexia.

High levels of inflammatory cytokines have been found to be associated with muscle wasting in an animal model using Walker-256 rats [37] and colon cancer 26 (C-26) [38]. In particular, tumor necrosis factor (TNF)-α and interleukin (IL) 1 and 6 were increased during cachexia, and administration of creatine or the Zhimu and Huangbai herb pair (ZBHP) was able to reduce muscle and adipose tissue wasting, suggesting that modulation of inflammatory signaling may be an interesting therapeutic target in cancer-related cachexia. A recent study in different mouse models based on the injection of C-26 murine adenocarcinomas or Lewis lung carcinoma (LLC) cells into BALB/c and C57BL/6 or Ager −/− (RAGE-null) mice reported that activation of the receptor for advanced glycation end-products (RAGE) by the S100 calcium-binding protein B (S100B) was able to induce muscle wasting and that the signaling pathway involved was the p38 MAPK/myogenin axis and signal transducer and activator of transcription (STAT) 3-dependent MyoD degradation [39]. Moreover, this study reported that muscle wasting, systemic inflammation, and the release of tumor-derived prokinetic factors were closely related to the chronic activation of RAGE that occurs under cancer conditions.

Decreased protein synthesis and increased proteolysis were also reported in C2C12 myotubes treated with TNF-α [40]. Interestingly, both treatment of C2C12 myotubes and intraperitoneal injection of TNF-α showed upregulation and activation of the extracellular signal-regulated kinase 1/2 (ERK1/2) and c-Jun N-terminal kinase (JNK). However, only inhibition of p38MAPKs reduced the upregulation of Atrogin1/MAFbx mRNA, suggesting that gene expression of Atrogin1/MAFbx mRNA is modulated by TNF-α via p38MAPKs signaling [40]. A mouse model of the fatal pediatric disease Duchenne muscular dystrophy (DMD) showed that TNF-mediated activation of JNK induces conformational changes in the insulin receptor substrate (IRS)-1, thereby disrupting downstream events after IGF-1/IGF-1 receptor binding [41]. As mentioned previously, IGF-1 controls muscle-mass homeostasis at multiple levels. Indeed, in a recent review, Yoshida and Delafontaine reported that the PI3K/Akt/mTOR and PI3K/Akt/GSK3β pathways contribute to protein synthesis in skeletal muscle through IGF-1 stimulation [42]. However, PI3K/Akt is also able to block E3 ubiquitin ligase, which inhibits FOXOs, thereby reducing protein degradation. Interestingly, the autophagy process is also negatively regulated by IGF-1 via mTOR and FOXO signaling with Akt being a major modulator. In addition, it has been suggested that IGF-1/Akt may reduce muscle wasting, inducing cytokines and myostatin signaling through inhibition of the nuclear factor kappa B (NF-κB) and Smad signaling pathways [42].

In vitro and in vivo experiments have shown that another member of the tumor necrosis factor superfamily may be involved in skeletal muscle regeneration, namely tumor necrosis factor-like weak inducer of apoptosis (TWEAK) and the receptor factor-inducible 14 (Fn14). Chronic administration and muscle-specific transgenic overexpression of TWEAK in mice showed cachectic effects, including low skeletal muscle weight and increased activity of UPS and NF-κB, whereas administration of anti-Fn14 monoclonal antibodies reduced tumor growth rate and progression to cachexia [43].

A study by Girgenrath and colleagues documented the expression of Fn14 in human primary mesenchymal stem cells and skeletal muscle myoblasts, and the activation of the NF-κB pathway after the TWEAK/Fn14 binding [44]. Moreover, TWEAK was able to induce cell proliferation in C2C12 myoblast culture, whereas terminal myogenesis was inhibited [44]. According to Pascoe and colleagues, the TWEAK/Fn14 effect could both positively and negatively influence skeletal muscle homeostasis depending on the different signaling pathways activated, including NFкB (canonical and non-canonical pathway), MAPK, and PI3K/Akt signaling [45].

It is well known that cachectic patients often develop behavioral changes such as anorexia that lead to reduced food intake, contributing to weight loss. A study by Torelli and colleagues, using an animal model with anorectic tumors, documented a reduction in anorexia symptoms after intraperitoneal injection of a soluble recombinant human TNF receptor [46]. Previous studies reported high levels of IL-1 mRNA and protein in cerebrospinal fluid from anorectic tumor-bearing rats [47,48]. Laviano and colleagues documented that intrahypothalamic injection of the IL-1 receptor antagonist improved anorexia symptoms [49]. According to van Norren and colleagues, chronic inflammation disrupts hypothalamic homeostasis, leading to dysregulation of the hypothalamic–pituitary–adrenal axis, resulting in anorexia and glucocorticoid-induced catabolic processes [50].

Evidence for IL-6 procachectic effects has been provided in animal models with tumors overexpressing IL-6. Administration of an antibody against IL-6R or genetic ablation of IL-6 significantly improved survival while reducing muscle wasting, whereas treatment with exogenous IL-6 resulted in muscle wasting in cancer-free mice [51,52,53]. Although several intracellular signaling pathways such as the janus kinase/signal transducer and activator of transcription (JAK-STAT)1/3, ERK MAPK, mTOR, and PI3K/Akt are associated with IL-6 release, it has been reported that IL-6/gp130-mediated activation of STAT3 is closely related to skeletal muscle wasting [54]. Interestingly, a study by Zimmers and colleagues showed that deletion of STAT3 did not result in skeletal muscle changes in animal models without cancer. In contrast, muscle mass and strength were preserved in cancer-related cachexia models, suggesting that STAT3 plays a more important role in disease than in the healthy state [55]. Moreover, deletion studies showed that reduced expression of CCAAT/enhancer-binding protein δ (C/EBPδ) resulted in low myostatin expression [56]. Human studies involving gastric, breast, and lung cancer patients (with or without cachexia) and healthy individuals showed a significant increase in IL-6 mRNA levels in cancer patients with cachexia compared to the other participant groups [57,58]. In addition, increased STAT3 pathway expression was reported [58]. These findings suggest that inflammatory cytokines modulate the protein turnover directly in skeletal muscle but can also indirectly impair muscle mass through anorexic stimuli.

## 4. Inflammation and Lipid Metabolism in Cancer-Related Cachexia

Chronic inflammation underlies several chronic diseases characterized by an impaired quality of life and is closely associated with poor outcome. The modulating effect of inflammatory mediators does not only affect the injured tissue and restricts the downstream cascade events to a well-defined area. Conversely, various organs are involved and play an important role in the homeostatic interplay of tissues.

A causative role of chronic inflammation in tumor development and progression has been reported since Virchow’s first observation in 1863, and to date, much of the literature has confirmed this role [59]. A recent review that focused on lipid droplets in cancer highlighted their critical importance in cancer cell survival [60]. As reported by Petan and colleagues, lipid droplet production depends on oxidative stress and nutrient deficiency. The function of lipid droplets is noteworthy as they regulate lipid trafficking and utilization for energy production, as well as damage control during oxidative stress and membrane biogenesis. Interestingly, lipid droplets also appear to be linked to the autophagy process to prevent lipotoxic phenomena [61].

A clinical study by de Matos-Neto and colleagues performed on patients with different cancers who were matched for diagnosis showed increased gene expression of TNF-α, IL-1β, monocyte chemoattractant protein-1 (MCP-1/CCL2), and CCL4 protein expression in the subcutaneous adipose tissue of cachectic cancer patients compared with those who were not cachectic [62]. The authors interpreted these results as increased crosstalk between cancer and adipose tissue after the onset of cachexia in cancer patients.

A study by Han and colleagues found that free fatty acids (FFA) were associated with serum levels of IL-6 and TNF-α in early stage and IL-6 levels in late-stage cachexia in gastric and colon cancer [63]. Moreover, lipolysis was strongly activated at all stages, whereas the white adipose tissue (WAT) browning occurred only at the late stage. Interestingly, blockade of the IL-6 receptor was able to inhibit WAT lipolysis and browning in the cachectic animal model.

A recent study focusing on possible gender differences between cachectic and non-cachectic cancer patients found that plasma concentrations of IL-6 and FFA were more elevated in cachectic patients than in the non-cachectic patients and that the female patients had higher levels of IL-6 and FFA compared with the male group [64].

Experimental studies on human myotubes cultured in conditioned media with highly cachectic cells (RXF393 cell line) reported an alteration in growth along with low expression levels of Myod1 and pan-myosin heavy chain (MHC) protein, although expression of myogenin, alpha-actinin, and fast-twitch MHC protein remained unchanged [65]. Treatment with RXF media resulted in the upregulation of NFкB, FoxO3, p38 MAPK, autophagosome, and fatty acid metabolism genes, while glycolytic genes were downregulated. In addition, metabolomic and lipidomic analysis revealed a large amount of triacylglycerides and fatty acids along with a high concentration of IL-1b, IL-6, IL-8, and TNF-α in RXF media. These results suggest an inflammation-induced alteration of glycolysis and free fatty acid metabolism. Inhibition of carnitine palmitoyltransferase-1 by etomoxir halted myofiber atrophy and decreased p38 MAPK signaling, which was the most activated stress-response pathway in this model. Thus, myotube growth and mitochondrial oxidative stress are negatively modulated by fatty acid oxidation.

In vitro and in vivo models of TNF-α-induced cachexia showed high ceramide levels during TNF-α treatment with increasing atrophy [66]. The authors documented that ceramide synthesis inhibitors were able to enhance protein synthesis, reduce proteolysis, and phosphorylate the transcription factor Foxo3, thereby decreasing the expression of the Atrogin-1 and LC3b genes. Notably, phospholipase D, S6K1, and Akt, all involved in the mTOR pathway, showed increased expression.

In a recent study, lipid profiles of cachectic cancer patients were compared with those of weight stable cancer patients [57]. In this study, different concentrations of saturated fatty acids (SFA), monounsaturated fatty acids (MUFA), and polyunsaturated fatty acids (PUFA) were found between the two groups of patients. Cachectic patients had increased levels of SFA (palmitic acid, stearic acid) and MUFA (oleic acid), while PUFA (linoleic acid, alpha-linolenic acid, gamma dihomolinoleic acid, and eicosapentaenoic acid) levels were reduced compared to the other group. In addition, plasma concentrations of the cytokines IL-6, TNF-α, and IL-8 were increased in the cachectic group.

The synthetic agonists GW9508 and TUG891 and the natural ligands ALA and DHA (α-linolenic acid and docosahexaenoic acid) of the free fatty acid receptors FFA1 and FFA4 showed a reduction in tumor weight and weight loss in LLC-bearing mice. A healthy effect on adipose tissue was also achieved by treatments with the selective FFA1 and FFA4 antagonists (GW1100 and AH7614, respectively) [67].

According to Hauck and Bernlohr, changes in the amount, composition, and localization of lipids can impair cellular functions [68]. The lipotoxicity paradigm has been proposed as an explanation for the negative effects associated with altered lipid metabolism. Specifically, an oxidative environment rich in reactive oxygen species (ROS) leads to mitochondrial production of hydroxyl radicals, which in turn trigger peroxidation of phospholipids and triglyceride lipid acyl chains, generating lipid radicals. Lipid peroxidation generates polyunsaturated lipid aldehydes and alters membrane structure depending on how well the short acyl chains of the peroxidized lipids penetrate the membranes. The reactive lipid aldehydes are involved in post-translational modification of proteins and cause carbonylation of amino acid residues of proteins with catalytic activity, leading to loss of function. In particular, 40% of the mitochondrial proteins interested in carbonylation have been reported to be involved in oxidative phosphorylation and thus associated with impaired mitochondrial function and ROS production [68].

Based on this evidence, one can speculate that chronic inflammation may disrupt lipid metabolism by increasing the production of reactive lipid aldehydes and may alter skeletal muscle homeostasis by carbonylation of the mitochondrial proteins.

In a contest characterized by altered glucose and lipidic metabolism, such as in cancer-related cachexia, several studies highlighted the potential therapeutic effects of peroxisome proliferator-activated receptors (PPARs) modulation [69]. The PPARs are members of the nuclear receptor family of transcription factors and their activation depends on the fatty acids and their downstream metabolites. In particular, they are implicated in peroxisomal and mitochondrial β-oxidation, microsomal ω-oxidation of fatty acids, glucose metabolism, energy expenditure, and inflammation [70,71]. It has been reported that three PPARs isotypes—namely PPARα (NR1C-1), PPARβ/δ (NR1C-2), and PPARγ (NR1C-3)— are expressed at different levels in different tissues [72]. PPARα is highly expressed in the liver, heart, brown adipose tissue, and kidney. Studies in animal models overexpressing PPARα reported the onset of glucose intolerance and insulin resistance along with 5′ adenosine monophosphate-activated protein kinase (AMPK) decreased activity, reduced glucose transporter GLUT4, and peroxisome proliferator-activated receptor-gamma coactivator 1 alpha (PGC1α) expression. These data were also confirmed by PPARα knockout mice studies [73]. The PPARβ/δ is predominantly expressed in skeletal muscle and in muscle satellite cells [74]. Studies in PPARβ/δ KO mice revealed a reduction in oxidative capacity of muscle with the development of obesity and diabetes [75]. Conversely, experimental use of PPARβ/δ agonist and transgenic animal model overexpressing PPARβ/δ showed increased glucose metabolism and fatty acid β-oxidation, small adipocytes, and a myofiber type switch from glycolytic to oxidative with an increase in mitochondrial number and activity [76,77]. The last isoform of PPARs, PPARγ, has been described as a key factor in organ lipid storage. In particular, it has been documented that the overexpression of PPARγ in the skeletal muscle of mice led to adiponectin production, reduction in myosteatosis, insulin sensitivity, and myofiber switching [78]. Conversely, PPARγ KO mice showed increased adipose tissue mass, glucose intolerance, and insulin resistance [79]. Thus, restoring the balance between glucose and lipid metabolism could lead to an improvement in cachectic symptoms.

## 5. Exercise Training Modulates Cytokines, Improving Cachexia Symptoms

There is a growing body of evidence linking exercise training to anti-inflammatory cytokine profiles [80]. A meta-analysis in cancer survivors found that TNF-α and C-reactive protein levels decrease in association with combined aerobic and resistance training [81]. Similar findings arise comparing the inflammatory cytokines profiles of elderly subjects after 6 months of aerobic or resistance exercise training [82]. In animal models with tumors, resistance training was shown to increase the IL-10/TNF-α ratio and circulating IL-10 levels [83]. As previously mentioned, elevated levels of IL-6 characterize the inflammatory profile of cachectic patients. However, it has been reported that endurance exercise greatly increases IL-6 plasma levels in healthy subjects and that cachectic patients with chronically high IL-6 levels showed a further increase after acute exercise [84]. Thus, the evidence for high IL-6 production in stimulated (temperature and O2) slow oxidizing muscle fibers, together with increased levels in arterial plasma after exercise, suggest a crucial role of exercise training in tissue crosstalk by modulating both pro- and anti-inflammatory responses [85,86].

Recently, endurance training has been considered for its known effectiveness on histological changes in skeletal muscle myofiber composition. As reported in endurance athletes, oxidative stress and changes in substrate availability trigger a metabolic shift from fast-glycolytic to slow-oxidative myofibers. A recent study involving experiments in C2C12 myotubes and mice showed an interesting relationship between free fatty acid availability and IL-13 signaling [87]. Remarkably, IL-13 does not appear to induce significant changes in metabolic gene expression in physiology, nor in IL-13-deficient mice at rest. In contrast, during acute exercise and endurance training, IL-13 signaling appears to be essential as evidenced by reduced fatty acid utilization and impaired mitochondrial biogenesis in an IL-13-deficient mice model. Furthermore, increased mitochondrial respiration, endurance performance, and glucose tolerance were found to be associated with IL-13/IL-13Ra1 binding and downstream activation of STAT3 signaling. Both IL-13 treatment in C2C12 myotubes and exercise training in mice increased phosphorylation levels of STAT3. The efficacy of exercise training in skeletal muscle homeostasis is also evidenced by its ability to enhance mitochondrial biogenesis involving peroxisome proliferator-activated receptor-gamma coactivator 1 alpha (PGC1α) [88]. It has been reported that PGC1α expression is increased in type I fibers and that the muscles of PGC1α transgenic mice rich in type II myofibers co-express a protein pattern typical of type I fibers. This coexpression of fibers was associated with increased resistance to fatigue.

Another factor that may play a role in the relationship between endurance training, mitochondrial biogenesis, and oxidative stress appears to involve a mitochondrial chaperone protein, namely heat shock protein 60 (Hsp60) [89]. A study addressing the role of Hsp60 in skeletal muscle showed a significant correlation between PGC1α, Hsp60 expression levels, and endurance training in mouse soleus muscle [90]. Fiber type specificity for Hsp60 was found with high expression in type IIa and type I fibers. The latter was increased by endurance training, which also increased the amount of Hsp60 protein [91]. In addition to high HSP60 levels, increased mitochondrial copy number and expression of three PGC1α isoforms were also detected [92]. The link between Hsp60 and PGC1α was established when Hsp60 was overexpressed in cultured myoblasts, resulting in the expression of the PGC1 1α isoform [90]. Subsequent studies have also shown that the different isoforms of PGC1α elicited by a single bout of endurance exercise are muscle type and sex-dependent [93].

**Figure 2:**
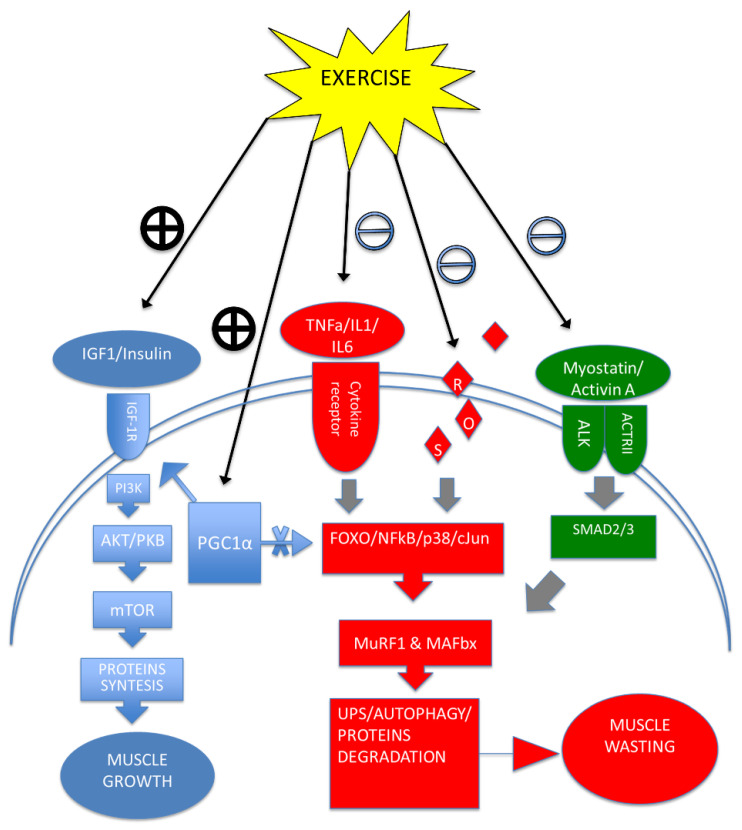
Exercise training regulates the balance between muscle growth and muscle wasting by modulating key protein synthesis and degradation processes. In blue, the direct effect of exercise training onto the IGF1 pathway and the central role of PGC1α in modulating both IGF1 and inflammatory pathways. In red and green, the negative modulation of exercise training on the inflammatory and myostatin/activin A pathways leading to muscle wasting. Positive modulation = 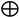; Negative modulation = 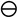.

These results support the idea that physical training could drive tissue cross-talk between skeletal muscle and adipose tissue through modulation of cytokines and reduce overall homeostatic dysfunction in cancer-related cachexia (Figure 2).

## 6. Conclusions

In summary, cancer cachexia is a multifactorial and organ-spanning syndrome in which the prominent clinical features result from a general disturbance of cross-talk between organs. The irreversible end-stage progression of several diseases that perpetuate the cachexia syndrome suggests that treatment of the main underlying cause of the syndrome, such as cancer in cancer-related cachexia, is the most important factor in stopping or at least delaying the onset. However, the progressive treatment of the various symptoms of the syndrome is a critical factor in improving quality of life. Moreover, it has been shown that the various attempts to treat this syndrome with a single agent do not resolve or alleviate the disease. Therefore, multimodal treatment seems to be the most likely approach, despite the known pharmacological interactions that may occur in clinical patients. The present review focused on the role of inflammatory cytokines in controlling cross-talk between organs and the resulting shift in metabolism leading to overall homeostatic dysfunction. In addition, physical exercise has been found to have a general effect on maintaining homeostasis in healthy life involving multiple organs and their metabolism. Therefore, studying the molecular effects of physical exercise could provide important information about the interactions between organs and the systemic mediators involved in the overall homeostasis of the body. Because of the wide-ranging effects of exercise training, from the body to the behavior and cognition of the individual, it seems to be able to improve the quality of life in this syndrome, which is also characterized by an activation of the stress hormone response from the preclinical stage to the non-reversible final stage of cancer-related cachexia. Future studies need to describe the contribution of each organ involved in this complex syndrome.

## Data Availability

Not applicable.
